# Development of High-Moisture Meat Analogues with Hemp and Soy Protein Using Extrusion Cooking

**DOI:** 10.3390/foods9060772

**Published:** 2020-06-11

**Authors:** Izalin Zahari, Ferawati Ferawati, Amanda Helstad, Cecilia Ahlström, Karolina Östbring, Marilyn Rayner, Jeanette K. Purhagen

**Affiliations:** 1 Department of Food Technology Engineering and Nutrition, Lund University, Naturvetarvägen 12, 223 62 Lund, Sweden; amanda.helstad@food.lth.se (A.H.); cecilia.ahlstrom@food.lth.se (C.A.); karolina.ostbring@food.lth.se (K.Ö.); marilyn.rayner@food.lth.se (M.R.); jeanette.purhagen@food.lth.se (J.K.P.); 2 Department of Chemistry and Biomedical Sciences, Linnaeus University, 392 31 Kalmar, Sweden; ferawati.ferawati@lnu.se

**Keywords:** high-moisture meat analogue, extrusion, hemp seed concentrate, soy protein isolate

## Abstract

The interest in plant-based products is growing in Western countries, mostly due to health and environmental issues that arise from the consumption and production of animal-based food products. Many vegan products today are made from soy, but drawbacks include the challenges of cultivating soy in colder climates such as northern Europe. Therefore, the present study investigates whether industrial hemp (*Cannabis sativa*) could substitute soy in the production of high moisture meat analogues (HMMA). A twin screw co-rotating extruder was used to investigate to what extent hemp protein concentrate (HPC) could replace soy protein isolate (SPI) in HMMAs. The substitution levels of HPC were 20 wt%, 40 wt% and 60 wt%. Pasting properties and melting temperature of the protein powders were characterized by Rapid Visco Analyzer (RVA) and Differential Scanning Calorimeter (DSC), respectively and the produced HMMA was analysed by determining the texture and colour attributes. The results showed that it is possible to extrude a mixture with up to 60% HPC. HPC absorbed less water and needed a higher denaturing temperature compared to SPI. Increasing the moisture content by 5% would have resulted in a reduction of hardness and chewiness. The lightness (L* value) was found to be significantly higher in SPI product and decreased in the mixture with higher HPC (*p* < 0.05).

## 1. Introduction

The Western diet includes a high proportion of products from animal origins, and the high consumption of meat is a subject of various debates on its impact on human health and the environment [1]. High consumption of meat is linked to an increased risk of cardiovascular disease, type 2 diabetes, and different types of cancer [1]. Moreover, livestock production is responsible for 14.5% of global greenhouse gas emissions [2]. A shift towards a more plant-based diet is suggested to be one possible solution to reduce climate impact and create a healthier lifestyle [3,4]. As a consequence, there is a tremendous market growth in the plant-based food sector, driven by a growing number of consumers adopting a vegan, vegetarian, or flexitarian lifestyle [5].

Plant-based alternative products range from dairy analogues to meat substitutes. The meat substitutes market is still relatively small, but is projected to increase in the future. Europe leads the meat substitutes market globally, with 40% of the total market [5], which is predicted to reach €2.4 billion in 2025 as compared to €1.5 billion in 2018 [5]. The market is also expected to increase in other regions, such as North America and Asia [5]. Tofu, tempeh, seitan, mycoprotein, and texturized vegetable protein are typical meat substitutes on the market. Another type of meat substitute, high-moisture meat analogue (HMMA), is not widely available on the market yet; thus, HMMA will be the focus of the present study. HMMA has a desirable chewy meat-like texture, which together with appropriate flavouring ingredients can offset the consumption of meat.

The vast majority of meat analogue products on the European market are made from imported soybean, while only a small proportion is made from other plant protein sources such as pea or mushroom. This import of raw material could potentially reduce the sustainable benefits of plant-based products. Therefore, other crops that could be cultivated over a range of latitudes, such as seeds from industrial hemp (*Cannabis sativa*) should be investigated. Optimization of the extrusion process in order to improve the texture of HMMA is one of the most challenging aspects when it comes to extrusion cooking. There have been many established extrusion processes and patents for HMMA production using soybean [6,7,8,9,10,11], but only a few studies have been carried out on formulations based on plant proteins other than soy [12,13,14].

The hemp plant has multifaceted benefits in many production sectors [15]. In Europe, hemp is mainly used as a source of fibre for biomass, textile, and the rope industry [15]. However, there is an increasing interest in using locally cultivated hemp seeds as a raw material for food production. Hemp seeds, the by-product from the fibre use for the construction industry, are rich in fats (25–35%, mostly unsaturated fatty acids), proteins (20–25%), dietary fibre (10–15%), vitamins, and minerals [16].

**Figure 1:**
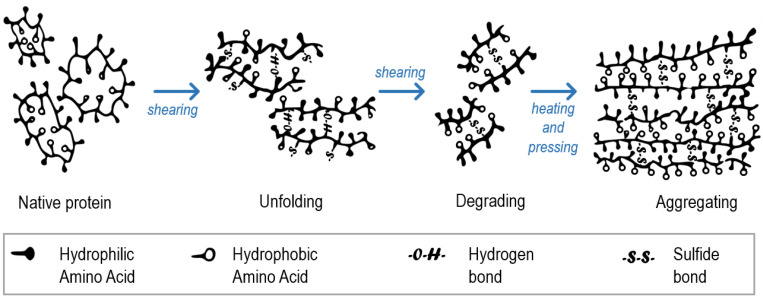
Changes in protein conformation during extrusion of HMMA. Modified illustration adopted from Zhang et al. [17].

Protein is the most important ingredient in the production of HMMA using extrusion technology. During extrusion, protein is denatured, unfolded, realigned and cross-linked, due to the shear, heat, pressure and cooling (Figure 1) achieved in the extruder and cooling die, respectively [17]. The final texture of HMMA is influenced by different types of protein bonds. Several factors determine the protein interactions such as protein type, pre-treatment and extrusion parameters [17].

Previous works have shown that hemp seed protein in particular, has a high protein quality and functionality [16,18,19]. However, no study uses hemp seed protein as a raw material for meat analogue production. Thus, this study was aimed to investigate the maximum ratio substitution of soy protein by hemp protein in the formulation of HMMA, as well as to study the characteristics of the HMMAs produced.

## 2. Materials and Methods 

**Figure 2:**
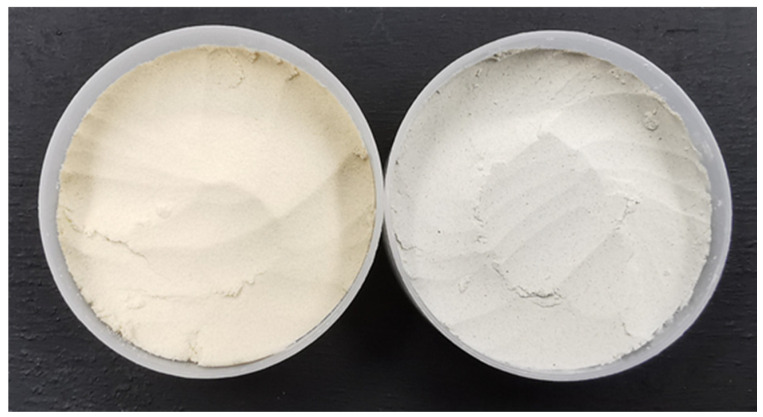
Soy protein isolate (left); and hemp protein concentrate (right) powder.

Soy protein isolate, SPI (91% protein, 1% carbohydrate, 3% fat, wet basis) and hemp protein concentrate, HPC (71% protein, 15% carbohydrate, 7% fat, wet basis), were obtained from Bulk Powders Company and Natures Organic Food Company, Sweden, respectively (Figure 2). Mixtures of hemp protein and soy protein (HPC:SPI) were prepared the same day as the extrusion trials according to the formulations (20:80, 40:60, 60:40) where the numbers represents wt% of each protein source.

### 2.1. Moisture Content

Moisture content of SPI and HPC powder sampled from packages as received from the suppliers, and the HMMA produced by extrusion were analysed. Three to five grams of sample was transferred into a metal container and put in an oven at 103 °C for at least 16 h until it reached constant weight using modification of AACC method 44-15A [20]. From the moisture content results of the raw materials, the mixtures’ moisture contents were calculated (Table 1). The moisture contents were important for the setting of solid and liquid dosing in the extruder software later to get the target moisture content for the final product. Extruded samples were collected immediately after the extrusion and were allowed to cool to room temperature where after the samples were sealed in plastic bags and placed in a freezer (−18 °C). The samples were thawed and cut into small pieces before the measurement using the same procedure for all samples.

### 2.2. Viscosity

The viscosity of both raw materials and mixtures of powders was measured using the standard AACC method 76-21 STD 1 [21] in the Rapid Visco Analyzer 4800 (Perten Instruments, Perkin Elmer, NSW, Australia). In brief, 3.50 g of sample was mixed with water (26.66 g) on 14% moisture basis as recommended by the manufacturer’s instruction. The samples were heated to 50 °C and stirred under a constant shear rate at 960 rpm for 10 s. The slurry was held at 50 °C for 50 s and then heated up to 130 °C, with temperature increasing at 12 °C/min. It was held at 130 °C for 2.5 min, and finally cooled to 50 °C. The pasting properties of each raw material and mixtures was measured at least in duplicate.

### 2.3. Thermal Properties

The thermal properties of the raw materials were determined using a Differential Scanning Calorimetry (DSC) instrument (Seiko Instruments Inc.-EXSTAR6000 DSC, Shizuoka, Japan). The instrument was calibrated with indium using an empty pan as a reference. 2 mg of protein powder was weighed into coated aluminium pan and hermetically sealed. Thereafter, three times the sample weight of MilliQ water was added. The pan was sealed and heated from 25 °C to 160 °C at a rate of 10 °C/min. Each sample was measured in triplicate and the data was stored and analysed using the DSC software (SII EXSTAR6000 Muse, Shizuoka, Japan). The melting temperature and the enthalpies were calculated from the thermograms based on the dry weight of the samples.

### 2.4. High-Moisture Extrusion Processing

HMMA products were processed using a laboratory, co-rotating twin-screw extruder (KETSE 20/40D, Brabender GmbH & Co.KG, Duisburg, Germany) using a fixed screw configuration (Table A1 in Appendix A) with a screw diameter of 20 mm. The extruder has four different temperature zones in the barrel which were maintained during the experiments at 40–60–80–100 °C. In the beginning, the chosen parameters considered in this study were concentration of HPC (0%, 20%, 40%, 60%), target moisture content (65%, 70%, 75%), temperature (40–120 °C) and screw speed (300–800 rpm). However, from our preliminary experiments, it was found that the screw speed of 800 rpm produced the most acceptable texture of HMMA products for all HPC mixtures formulations whereas for SPI alone, we were only able to extrude soy protein using 500 rpm at 70% and 75% of target moisture content. Thus, this experiment was carried out using eleven formulations as summarized in Table 2. The solid dosing (0.8–1.1 kg/h) and liquid dosing (1.88–2.36 kg/h) was calculated from the moisture content of the raw materials, while the target moisture content of the product and the total mass flow (3 kg/h) was set using the complementary software from Brabender GmbH & Co.KG. The cooked mixture was pushed through a long flat shaped cooling die with dimensions of 25 × 7 × 300 mm (W × H × L) which was cooled by a stream of water from the outside. The cooling process increases the viscosity of the mixture, thus inducing shear that generates a layered and fibrous structure. Extruded products were manually cut at the end of the cooling die and were allowed to cool at room temperature before the material was transferred to a sealed plastic bag where it was kept frozen at −18 °C prior to further analysis.

### 2.5. Texture Properties

**Figure 3:**
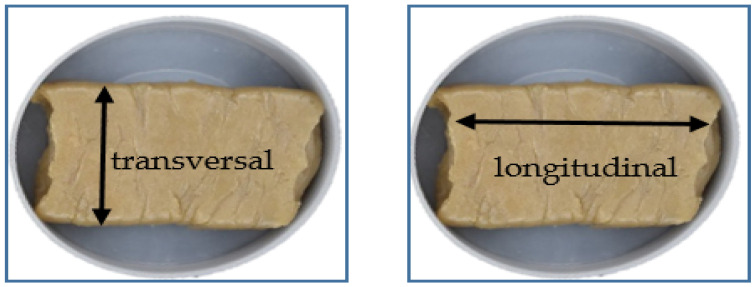
Transversal and longitudinal cutting directions of samples.

Texture profile analysis (TPA), cutting test, and tensile strength were carried out to evaluate the overall texture of the extruded products. The measurements were performed using a texture analyser (TVT-300XP, Perten Instruments AB, Hägersten, Sweden) [13,22]. For TPA, samples size of 2 × 2 cm with 7 mm thickness were compressed by 2 mm using a cylindrical probe (Ø 18 mm). Hardness, springiness, resilience and chewiness of the samples were determined. Hardness is a measurement of how hard the product is and can be determined by the maximum force of the first compression [23]. Springiness shows how well the product returns to its original structure after the first compression, while resilience describes how well the product regains its original height after the compression [23]. Chewiness shows how much work is needed to chew the product to achieve the texture that is suitable for ingestion [24]. Other parameters such as cutting and tensile strength could be linked to the quality of meat, for instance, describing the tenderness [25]. A minimum of three measurements for each sample was performed. For the cutting test, two types of cutting strength were measured: transversal and longitudinal. Samples of 2 × 2 cm (transversal) and 5 × 2 cm (longitudinal) with 7 mm thickness were cut 5 mm deep using a knife blade (height 117 mm) at a speed of 2 mm/s. Transversal cutting was done in the direction of the width of the sample whereas longitudinal cutting was done in the direction of the length of the sample (Figure 3). For tensile strength, a sample of 2 × 12 cm was prepared. The sample was attached to the rig and stretched 30 mm at a speed of 1 mm/s and the break force (g) was measured. 

### 2.6. Colour Determination and Appearance of HMMA

The colour of the produced HMMA products was measured using a colorimeter (Konica Minolta CR-400, Osaka, Japan). The calibration was performed with a white calibration tile before the measurement. The CIE-Lab parameters were expressed as a value of L* (lightness), a* (redness or greenness) and b* (yellowness or blueness). The measurements were performed in triplicate for each sample at randomly selected locations. For the appearance, images were acquired by a rigged camera (Nikon D3300, AF-P DX 18-55/3.5-5.6G, Nikon Corporation, Tokyo, Japan) in a photo box with black walls and four light sources pointed to the sample to make high quality pictures of the HMMA products. 

### 2.7. Statistical Analysis

All experimental work was carried out on freshly prepared samples, in at least duplicates, and reported as means ± standard deviations of the measurements. All analyses were performed using Microsoft Office Excel 2010 and Minitab 16.2.1 statistical software (Minitab Inc., Pennsylvania, PA, USA). Tukey´s test was used to identify the differences in each treatment at *p* < 0.05.

## 3. Results and Discussion

### 3.1. Moisture Content

The moisture content of all HMMA products was analysed to investigate if it corresponded to the target moisture content with the solid and liquid dosing. It was found that the differences between the target and measured moisture content varied between −4.38% to 4.76%. The measured moisture content of 0% HPC (100% SPI) sample differed more (74.38 and 76.06) than the targeted moisture value (70% and 75%), respectively, whereas other mixture samples with HPC differed less than the targeted moisture values. The 20% HPC sample had the most exact value compared with the target, followed by 40% and 60%. This result proved that there was no significant variation between them based on the accuracy of the method. The variations in moisture content could possibly be due to an uneven flow rate of powder from the hopper during the extrusion process.

### 3.2. Pasting Properties

**Figure 4:**
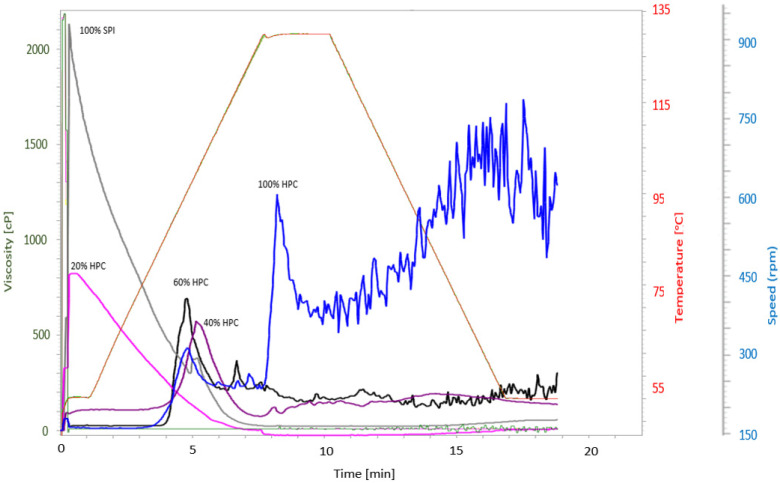
RVA curves for the raw materials and mixtures. (100% SPI: grey, 100% HPC: blue, 20% HPC: pink, 40% HPC: purple, 60% HPC: black.).

The Rapid Visco Analyzer (RVA) was used to measure several parameters related to the pasting properties of the raw materials used. Obvious differences were found between the samples when it comes to their behaviour during heating and cooling in an excess of water. From Figure 4, the SPI (grey curve) showed the highest peak viscosity among all samples which indicates a good paste texture and high water-binding capacity. This characteristic could provide excellent texture which is desirable in HMMA products [26]. Despite that, HPC alone showed a rather inconsistent behaviour which was also found in the 40% (purple curve) and 60% (black curve) HPC-SPI mixtures. At the end of the analysis, it was observed that the HPC sample formed spheres inside the container which might alter the result. The spheres hinder the paddle in the rotation causing this inconsistence behaviour. According to some researchers, the interaction of starch and fatty acids may also contribute to a new complex, thus affecting some modification and texture stability of the final product as previously reported by others [27,28]. Pasting temperature is the minimum temperature required to cook a sample [28], and it was found to vary from 53.8 °C to 90.8 °C for HPC-SPI mixtures compared to the SPI sample (50.2 °C). This indicates that cold swelling takes place directly for pure SPI and 20% HPC mixture (pink curve). SPI and the 20% HPC mixture required the shortest time to reach maximum viscosity (0.4 min and 0.3 min, respectively). It was noted that for the samples with higher degree of hemp substitution, a longer time was required (8.2 min for 100% HPC). This means that HPC needs more time and higher temperatures to bind water and denature before reaching the maximum viscosity compared to the SPI. The maximum viscosity was reached due to the reduction of protein solubility as a result of denaturation of native proteins [13]. Overall, these results indicate that the substitution of HPC had a readily observable influence on pasting behaviour.

### 3.3. Thermal Properties

**Figure 5:**
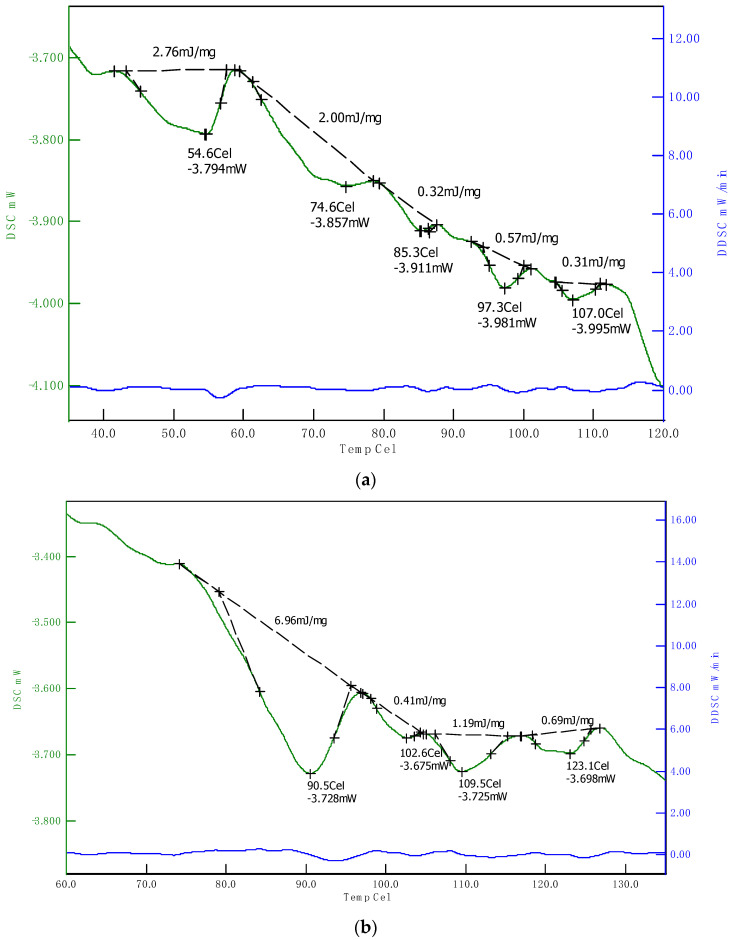
DSC thermograms for (**a**) SPI; and (**b**)HPC.

Different melting temperatures were observed for SPI and HPC in the DSC analysis, due to different protein contents. There were one to four endothermic peaks identified in HPC, whereas three to five endothermic peaks were observed in the SPI. The proteins in the HPC had overall higher melting temperatures (90.3–123.1 °C) as compared with SPI (54.6–107.0 °C) (Figure 5). This indicates that the cooking zone in the extruder with the highest temperature should be set to at least 123.1 °C if all proteins in the HPC should denature during extrusion. One main peak was detected in HPC which probably corresponds to Edestin, a storage protein in the hemp seed. It was found in all samples with high enthalpies (5.53–7.25 mJ/mg) [16,19]. For SPI, peaks for 7 S and 11 S globulins were identified, but with relatively lower enthalpies (0.17–2.00 and 0.57–0.90 mJ/mg, respectively) [29].

### 3.4. Texture Properties

**Figure 6:**
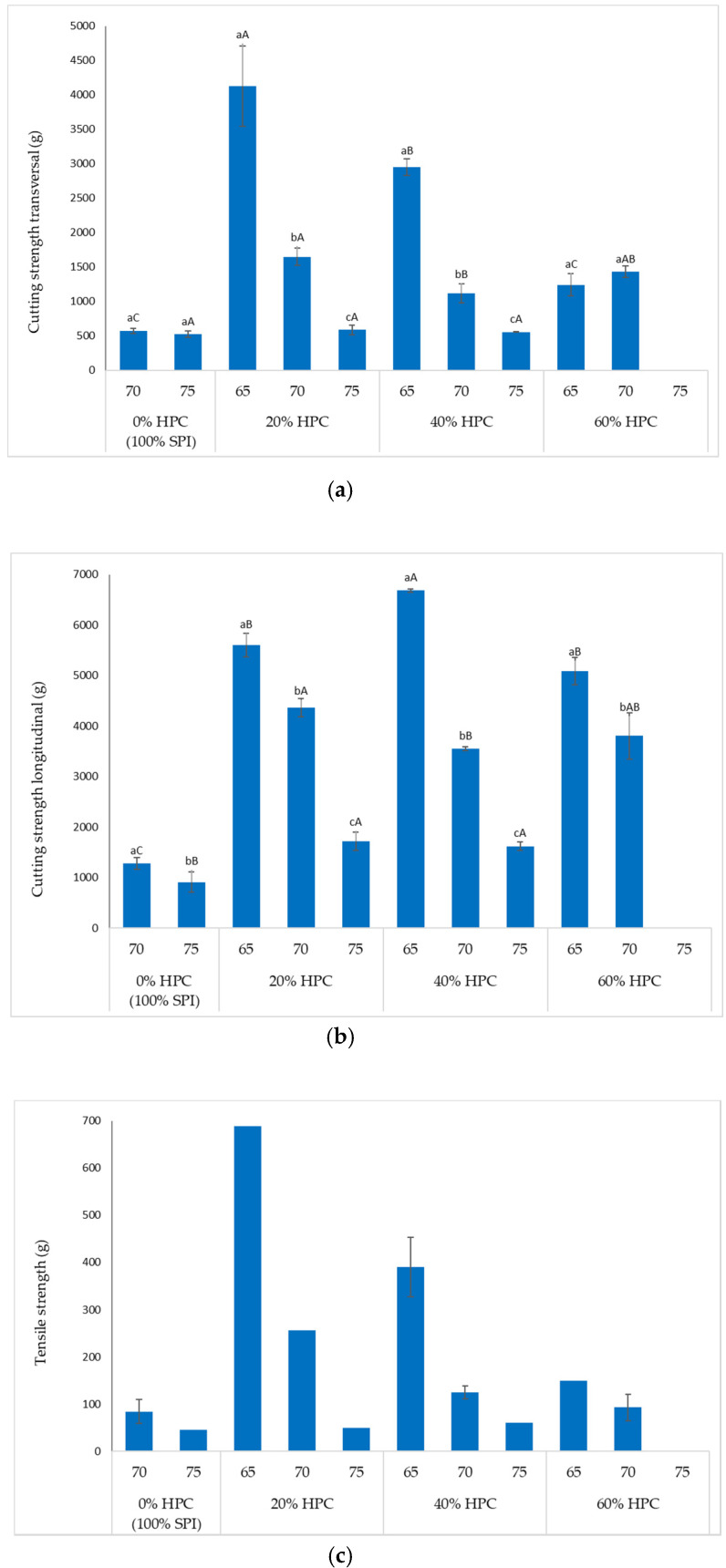
(**a**) Transversal cutting strength; (**b**) longitudinal cutting strength; (**c**) tensile strength of different HMMA formulations at each target moisture contents. Bars represent the mean ± standard deviation of triplicate measurements. Different lowercase letters indicate significant differences (Tukey’s test, *p* < 0.05) within the same HMMA formulation. Different uppercase letters indicate a significant difference between different HMMA formulations. Bars without standard deviation represent only one measurement.

Samples from all HMMA formulations were analysed in terms of texture profiles. All HMMA made from 100% SPI showed no significant differences (*p* < 0.05) for hardness, springiness, and chewiness at 70% and 75% target moisture content, except for the resilience, longitudinal cutting and tensile strength (Table 3 and Figure 6). Whereas, the substitution of SPI with different percentage of HPC had no significant effect (*p* < 0.05) on the springiness of HMMA at all targeted moisture contents. The resilience value ranged from the lowest (0.48) in 100% SPI at 75% moisture content to the highest (1.66) in 40% HPC with 70% moisture content. This means that HMMA made from 40% HPC and 70% moisture content had the highest elasticity when compared with other samples. Furthermore, springiness and resilience value of 40% and 60% HPC were found to not correlate with the moisture content, which may be due to the higher number of air cells in meat analogue structures, thus leading to lower integrity and difference distribution of fibrous texture [22]. It also might be due to the uneven flow rate from the hopper, which could have led to incompletely homogenous product during protein texturization. On the other hand, the hardness and chewiness of HMMA made from HPC were strongly affected by the target moisture content. Increasing the moisture content by 5% would have resulted in significant reduction (*p* < 0.05) of hardness and chewiness. This observation is in agreement with others who reported an increase in hardness and chewiness value as the moisture content decreased in HMMA made from soy protein [7]. Important to note is that the HMMA made from 60% HPC with target moisture content of 75% had a very soft and brittle texture and therefore no texture analysis could be performed on those samples. 

The results for the transversal cutting depended on where on the sample the cut was performed due to the structure of the sample. The longitudinal cut gave a more complete overview of the fibre structure because the cutting was performed over the fibres rather than cutting between the fibres as for the transversal cutting. The Tukey´s test indicated that moisture content had a significant effect (*p* < 0.05) on transversal and longitudinal cutting strength as well as the tensile strength values (Figure 6). An increase in moisture content resulted in HMMA products that were easier to cut (cutting strength) and break (tensile strength). The same effect of moisture increase had been reported before which resulted in a reduction of cutting force of HMMA made from lupin protein isolate [30]. Several authors have also argued that higher moisture content affects the viscosity and temperature of the mixture in the extruder, which might lead to incomplete denaturation and texturizing of the protein [7,30]. Almost all texture properties of the HMMA samples made from 100% SPI were the lowest when compared to other samples.

### 3.5. Colour Determination and Appearance of HMMA

Colour measurement of all samples was performed, and L*, a*, and b* values are reported in Table 4. In general, it can be seen in Figure 2 that the original colour of the SPI powder is darker than the HPC powder. However, after the extrusion process, the HMMA produced from SPI became lighter and higher substitution levels of HPC produced HMMA product with darker colour. The colour changes occurred due to several reactions during the extrusion process, including the Maillard reaction, caramelization, hydrolysis, and a nonenzymatic reaction, such as the degradation of pigments [31]. The lightness (L* value) was found to be significantly higher in SPI product and decreased with higher substitution of HPC (*p* < 0.05). The same trend was observed for a* and b* values, where samples with a higher substitution of hemp protein gave more redness and less yellowness compared to 100% soy. It can also be seen that at all of the substitution levels of HPC:SPI mixtures, the L* values of the product at 70% and 75% of moisture content were not statistically different (lowercase letters) (*p* < 0.05). On the other hand, when analysing each formulation (20%, 40% and 60% HPC), most of the results show significant differences between the moisture content for all the colour values, except for a* and b* values of 60% and 20% mixtures, respectively.

**Figure 7:**
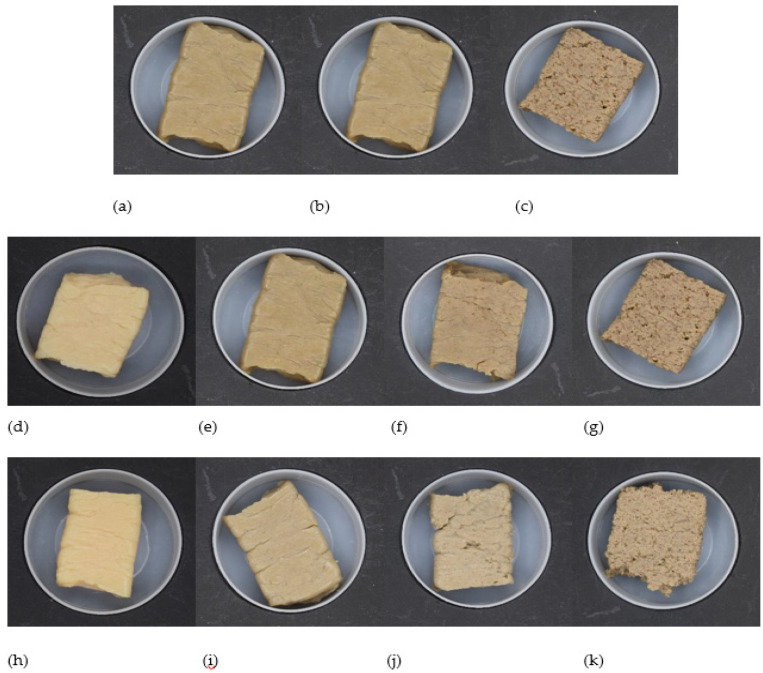
Images of extruded meat analogues with different formulations: (**a**–**c**) 20%, 40%, 60% HPC at 65% target moisture; (**d**–**g**) 0%, 20%, 40%, 60% HPC at 70% target moisture; (**h**–**k**) 0%, 20%, 40%, 60% HPC at 75% target moisture.

In terms of the appearance of the product, it can be seen from Figure 7 that the meat analogue from pure SPI was more compact, with a clear fibrous structure as compared with the other products. This may be due to the higher protein composition in SPI (90% compared to 70% in HPC) which could create the protein cross-linking reaction and formation of disulphide bonds during the extrusion process [7]. High content of methionine and cystine residues may also resulted in increasing the formation of covalent disulphide bonds between individual molecules, thus increasing the extent of aggregation [19]. While with the addition of HPC, with less protein content and higher content of other components such as carbohydrate and fat could affect the internal reaction of the melt in the extruder, resulting in a soft and less compact product [32,33]. It was found that the reordering of the segments of fibres and other ingredients in the starch matrix and complex formulation also resulted in different effects which could modify the physical properties of extruded products [32]. Figure 7 presents the different extruded products from each formulation at 65%, 70% and 75% moisture content. The formulation with 60% HPC produced a meat analogue with dark spots and liquid phase separation with foam covering the surface, which might be due to the undenatured protein of HPC. As can be seen from DSC and RVA results, it is recommended to increase cooking temperature and retention time as hemp needs higher temperature to induce protein denaturation. In addition, hemp protein probably needs more kneading elements in the screw configuration in order to increase the retention time and to develop a more laminar fibre structure.

## 4. Conclusions

Based on this study, it was possible to substitute soy protein with hemp protein in the meat analogue formulation. Hemp protein content (20%, 40%, 60%) at the different targeted moisture contents (65%, 70%, 75%) and a screw speed of 800 rpm had a pronounced effect on the texture and colour of the final product. HPC could therefore be a promising novel material to be included into extruded products and this study shows that the resulting meat analogue gave a comparable texture to SPI alone, and that soy protein could be substituted by hemp protein by up to 60%. More studies are needed in order to fully understand the complex extrusion process and the formulation matrix including the nutritional composition and consumer acceptance towards the final product. 

## Figures and Tables

**Table 1:** Moisture content data for the raw materials of the mixtures.

Raw Material	Moisture Content (%)
100% SPI	6.39 ± 0.03
100% HPC	8.02 ± 0.07
**Mixtures (HPC:SPI) (%)**	**Calculated Moisture Content (%)**
20:80	6.72
40:60	7.04
60:40	7.37

**Table 2:** Extrusion parameters involved in the screening and selected formulations of HMMA.

Formulation	Screw Speed (rpm)	Target Moisture Content (%)	Temperature (°C) (Zone 1–2–3–4)	Selected Trial
0% HPC (100% SPI)	500	70	40–60–80–100	√
		75		√
20% HPC	800	65	40–60–80–100	√
		70		√
		75		√
	600	75		
		80		
	400	75		
	300	75		
40% HPC	800	65	40–60–80–100	√
		70		√
		75		√
60% HPC	800	60	40–60–80–100	
		62.5		
		65		√
		70		√
		75		√
		65	60–80–100–120	

**Table 3:** Texture profile analysis result of HMMA at different formulations and target moisture content ^1^.

Formula	Moisture Content (%)	Hardness (g)	Springiness	Resilience	Chewiness (g)
0% HPC (100% SPI)	70	527 ± 33 ^aC^	0.99 ± 0.01 ^aA^	0.51 ± 0.01 ^aB^	459 ± 34 ^aC^
	75	545 ± 66 ^aB^	0.99 ± 0.00 ^aA^	0.48 ± 0.01 ^bB^	465 ± 44 ^aC^
20% HPC	65	3444 ± 186 ^aA^	0.88 ± 0.14 ^aA^	1.59 ± 0.88 ^aA^	2944 ± 123 ^aA^
	70	1911 ± 18 ^bA^	0.95 ± 0.06 ^aA^	0.55 ± 0.02 ^aB^	1630 ± 19 ^bA^
	75	798 ± 21 ^cA^	0.96 ± 0.05 ^aA^	0.52 ± 0.01 ^aA^	670 ± 8 ^cB^
40% HPC	65	3275 ± 76 ^aA^	0.91 ± 0.14 ^aA^	0.57 ± 0.03 ^bA^	2692 ± 71 ^aB^
	70	1700 ± 66 ^bB^	1.19 ± 0.12 ^aA^	1.66 ± 0.26 ^aA^	1435 ± 44 ^bB^
	75	851 ± 42 ^cA^	0.97 ± 0.04 ^aA^	0.51 ± 0.04 ^bAB^	721 ± 29 ^cA^
60% HPC	65	2640 ± 84 ^aB^	1.02 ± 0.19 ^aA^	1.20 ± 0.60 ^aA^	2128 ± 34 ^aC^
	70	1719 ±116b ^bB^	0.93 ± 0.04 ^aA^	0.52 ± 0.07 ^aB^	1410 ± 59 ^bB^
	75	Too soft and brittle to measure			

^1^ All values are presented as mean ± standard deviation. Different lowercase letters indicate a significant difference (Tukey’s test, *p* < 0.05) between different target moisture at the same HMMA formulation, and different uppercase letters indicate a significant difference between different HMMA formulations at the same target moisture content.

**Table 4:** Result of L*, a* and b* using colorimeter with a short description of each product ^1^.

Sample	Colorimeter			Visual Appearance
Moisture Content (%)	L*	a*	b*	
0% HPC (100% SPI)				
70	61.67 ± 0.25 ^A^	−0.24 ± 0.07 ^A^	13.48 ± 0.43 ^A^	Light colour, compact
75	62.55± 0.21 ^A^	−0.29 ± 0.19 ^A^	13.00 ± 0.53 ^A^	Light colour, compact
20% HPC				
65	46.19 ± 0.68 ^aB^	0.84 ± 0.08 ^aB^	8.45 ± 0.23 ^aB^	Compact
70	46.84 ± 0.68 ^bC^	0.69 ± 0.15 ^bC^	9.20 ± 0.42 ^bB^	Compact
75	48.87 ± 0.29 ^bC^	0.30 ± 0.09 ^bC^	8.78 ±0.14 ^abB^	Less compact
40% HPC				
65	45.92 ± 1.30 ^aC^	1.64 ± 0.35 ^aD^	9.22 ± 1.02 ^aB^	Compact, dark spot
70	48.65 ± 0.52 ^bB^	1.73 ± 0.11 ^aD^	10.90 ± 0.45 ^aC^	Compact
75	50.96 ± 0.78 ^bD^	0.95 ± 0.15 ^bC^	10.34 ± 0.40 ^aC^	Pale, less compact
60% HPC				
65	45.43 ± 0.33 ^aC^	2.34 ± 0.20 ^aE^	7.98 ± 0.72 ^aB^	Less compact
70	50.42 ± 0.56 ^bBD^	2.29 ± 0.04 ^aE^	10.11 ± 0.47 ^bB^	Less compact
75	49.78 ± 1.01 ^bD^	2.23 ± 0.15 ^aE^	9.56 ± 0.41 ^bC^	Came out foamy

^1^ All L*, a*, b* values are presented as mean ± standard deviation. Different lowercase letters indicate a significant difference (*p* < 0.05) between different moisture content with the same HPC content. Different uppercase letters indicate a significant difference between all HMMA formulations including SPI.

## Appendix A

**Table A1:** Screw configuration used for extrusion of HMMA in this study ^1^.

Screw Configuration for Meat Analogue Product			
1.	SE-30/30A	18.	SE-20/20R
2.	SE-30/30R	19.	SE-20/20R
3.	SE-30/30R	20.	SE-20/20R
4.	SE-30/30R	21.	KBW-45/5/20/R
5.	SE-30/30R	22.	KBW-45/5/30/R
6.	SE-20/20R	23.	SE-20/10L
7.	SE-20/20R	24.	SE-30/30R
8.	SE-20/20R	25.	SE-20/10L
9.	SE-20/20R	26.	SE-30/30R
10.	SE-30/30R	27.	SE-20/10L
11.	SE-30/30R	28.	KBW-45/5/30/R
12.	SE-20/20R	29.	KBW-45/5/20/L
13.	KBW-45/5/20/R	30.	SE-30/15R
14.	SE-30/30R	31.	SE-20/20R
15.	SE-30/30R	32.	SE-20/20R
16.	SE-30/30R	33.	SE-20/20R
17.	SE-20/20R	34.	SE-30/30R

^1^ SE—screw element; KBW—kneading block element.

## References

[1] Richi E.B., Baumer B., Conrad B., Darioli R., Schmid A., Keller U. (2015). Health Risks Associated with Meat Consumption: A Review of Epidemiological Studies. Int. J. Vitam. Nutr. Res..

[2] Gerber P., Steinfeld H., Henderson B., Mottet A., Opio C., Dijkman J., Falcucci A., Tempio G. (2013). Tackling Climate Change through Livestock—A Global Assessment of Emissions and Mitigation Opportunities.

[3] Röös E., Bajželj B., Smith P., Patel M., Little D., Garnett T. (2017). Greedy or Needy? Land Use and Climate Impacts of Food in 2050 under Different Livestock Futures. Glob. Environ. Chang..

[4] EAT-Lancet Commission (2019). Healthy Diets from Sustainable Food Systems: Food Planet Health.

[5] Deloitte (2019). Plant-Based Alternatives-Driving Industry M&A Contents.

[6] Liu K.S., Hsieh F.H. (2007). Protein-Protein Interactions in High Moisture-Extruded Meat Analogs and Heat-Induced Soy Protein Gels. JAOCS J. Am. Oil Chem. Soc..

[7] Lin S., Huff H.E., Hsieh F. (2000). Texture and Chemical Characteristics of Soy Protein Meat Analog Extruded at High Moisture. J. Food Sci..

[8] Lin S., Huff H.E., Hsieh F. (2002). Extrusion Process Parameters, Sensory Characteristics, and Structural Properties of a High Moisture Soy Protein Meat Analog. J. Food Sci..

[9] Cheftel J.C., Kitagawa M., Queguiner C. (1992). New Protein Texturization Processes by Extrusion Cooking at High Moisture Levels. Food Rev. Int..

[10] Pietsch V.L., Bühler J.M., Karbstein H.P., Emin M.A. (2019). High Moisture Extrusion of Soy Protein Concentrate: Influence of Thermomechanical Treatment on Protein-Protein Interactions and Rheological Properties. J. Food Eng..

[11] Chiang J.H., Loveday S.M., Hardacre A.K., Parker M.E. (2019). Effects of Soy Protein to Wheat Gluten Ratio on the Physicochemical Properties of Extruded Meat Analogues. Food Struct..

[12] Osen R., Toelstede S., Eisner P., Schweiggert-Weisz U. (2015). Effect of High Moisture Extrusion Cooking on Protein-Protein Interactions of Pea (*Pisum sativum* L.) Protein Isolates. Int. J. Food Sci. Technol..

[13] Osen R., Toelstede S., Wild F., Eisner P., Schweiggert-Weisz U. (2014). High Moisture Extrusion Cooking of Pea Protein Isolates: Raw Material Characteristics, Extruder Responses, and Texture Properties. J. Food Eng..

[14] Schreuders F.K.G., Dekkers B.L., Bodnár I., Erni P., Boom R.M., van der Goot A.J. (2019). Comparing Structuring Potential of Pea and Soy Protein with Gluten for Meat Analogue Preparation. J. Food Eng..

[15] Young E.M. (2005). Revival of Industrial Hemp: A Systematic Analysis of the Current Global Industry to Determine Limitations and Identify Future Potentials within the Concept of Sustainability. Master’s Thesis.

[16] Wang Q., Xiong Y.L. (2019). Processing, Nutrition, and Functionality of Hempseed Protein: A Review. Compr. Rev. Food Sci. Food Saf..

[17] Zhang J., Liu L., Liu H., Yoon A., Rizvi S.S.H., Wang Q. (2019). Changes in Conformation and Quality of Vegetable Protein during Texturization Process by Extrusion. Crit. Rev. Food Sci. Nutr..

[18] Raikos V., Neacsu M., Russell W., Duthie G. (2014). Comparative Study of the Functional Properties of Lupin, Green Pea, Fava Bean, Hemp, and Buckwheat Flours as Affected by PH. Food Sci. Nutr..

[19] Tang C.H., Ten Z., Wang X.S., Yang X.Q. (2006). Physicochemical and Functional Properties of Hemp (*Cannabis sativa* L.) Protein Isolate. J. Agric. Food Chem..

[20] AACC (2000). AACC Method 44-15A. Moisture—Air-Oven Method. AACC Approved Methods of Analysis.

[21] AACC (1997). AACC Method 76-21.02. General Pasting Method for Wheat or Rye Flour or Starch Using the Rapid Visco Analyser. AACC Approved Methods of Analysis.

[22] Samard S., Gu B.Y., Ryu G.H. (2019). Effects of Extrusion Types, Screw Speed and Addition of Wheat Gluten on Physicochemical Characteristics and Cooking Stability of Meat Analogues. J. Sci. Food Agric..

[23] Texture Profile Analysis. https://texturetechnologies.com/resources/texture-profile-analysis#tpa-measurements.

[24] Da Silva V.B., da Costa M.P., Grumezescu A.M., Holban A.M. (2019). Influence of Processing on Rheological and Textural Characteristics of Goat and Sheep Milk Beverages and Methods of Analysis. Processing and Sustainability of Beverages.

[25] Morey A., Owens C.M., Petracci M., Berri C. (2017). Methods for Measuring Meat Texture. Poultry Quality Evaluation: Quality Attributes and Consumer Values.

[26] Alamu E.O., Maziya-Dixon B., Dixon A.G. (2017). Evaluation of Proximate Composition and Pasting Properties of High Quality Cassava Flour (HQCF) from Cassava Genotypes (Manihot Esculenta Crantz) of β-Carotene-Enriched Roots. LWT.

[27] Bemiller J.N. (2011). Pasting, Paste, and Gel Properties of Starch-Hydrocolloid Combinations. Carbohydr. Polym..

[28] Cozzolino D. (2016). The Use of the Rapid Visco Analyser (RVA) in Breeding and Selection of Cereals. J. Cereal Sci..

[29] Kitabatake N., Tahara M., Dol E. (1990). Thermal Denaturation of Soybean Protein at Low Water Contents. Agric. Biol. Chem..

[30] Palanisamy M., Töpfl S., Berger R.G., Hertel C. (2019). Physico-Chemical and Nutritional Properties of Meat Analogues Based on Spirulina/Lupin Protein Mixtures. Eur. Food Res. Technol..

[31] Camire M.E., Camire A.L., Krumhar K. (1990). Chemical and Nutritional Changes. Crit. Rev. Food Sci. Nutr..

[32] Santillán-Moreno A., Martínez-Bustos F., Castaño-Tostado E., Amaya-Llano S.L. (2011). Physicochemical Characterization of Extruded Blends of Corn Starch-Whey Protein Concentrate-Agave Tequilana Fiber. Food Bioprocess Technol..

[33] Malav O.P., Talukder S., Gokulakrishnan P., Chand S. (2015). Meat Analog: A Review. Crit. Rev. Food Sci. Nutr..

